# Medulloblastoma and ependymoma cells display increased levels of 5-carboxylcytosine and elevated *TET1* expression

**DOI:** 10.1186/s13148-016-0306-2

**Published:** 2017-02-13

**Authors:** Ashley Ramsawhook, Lara Lewis, Beth Coyle, Alexey Ruzov

**Affiliations:** 1 Division of Cancer and Stem Cells, School of Medicine, Centre for Biomolecular Sciences, University of Nottingham, University Park, Nottingham, NG7 2RD UK; 2 Children’s Brain Tumour Research Centre, School of Medicine, QMC, University of Nottingham, Nottingham, NG7 2UH UK

**Keywords:** DNA methylation, 5-hydroxymethylcytosine, 5-carboxylcytosine, Paediatric brain tumours, Medulloblastoma, Ependymoma, Immunohistochemistry

## Abstract

**Background:**

Alteration of DNA methylation (5-methylcytosine, 5mC) patterns represents one of the causes of tumorigenesis and cancer progression. Tet proteins can oxidise 5mC to 5-hydroxymethylcytosine (5hmC), 5-formylcytosine and 5-carboxylcytosine (5caC). Although the roles of these oxidised forms of 5mC (oxi-mCs) in cancer pathogenesis are still largely unknown, there are indications that they may be involved in the mechanisms of malignant transformation. Thus, reduction of 5hmC content represents an epigenetic hallmark of human tumours, and according to our recent report, 5caC is enriched in a proportion of breast cancers and gliomas. Nevertheless, the distribution of oxi-mCs in paediatric brain tumours has not been assessed.

**Findings:**

Here, we analyse the global levels and spatial distribution of 5hmC and 5caC in four brain tumour cell lines derived from paediatric sonic hedgehog (SHH) pathway-activated medulloblastomas (Daoy and UW228-3) and ependymomas (BXD-1425EPN and DKFZ-EP1NS). We show that, unlike HeLa cells, the paediatric tumour cell lines possess both 5hmC and 5caC at immunochemically detectable levels and demonstrate that both modifications display high degrees of spatial overlap in the nuclei of medulloblastomas and ependymomas. Moreover, although 5hmC levels are comparable in the four brain tumour cell lines, 5caC staining intensities differ dramatically between them with highest levels of this mark in a subpopulation of DKFZ-EP1NS cells. Remarkably, the 5caC enrichment does not correlate with 5hmC levels and is not associated with alterations in thymine DNA glycosylase (*TDG*) expression in SHH medulloblastoma and ependymoma cell lines but corresponds to elevated levels of *TET1* transcript in UW228-3 and DKFZ-EP1NS cells.

**Conclusions:**

We demonstrate that both 5caC enrichment and elevated *TET1* expression are observed in SHH medulloblastomas and ependymomas. Our results suggest that increased Tet-dependent 5mC oxidation may represent one of the epigenetic signatures of cancers with neural stem cell origin and, thus, may contribute to development of novel approaches for diagnosis and therapy of the brain tumours.

## Findings

### Background

Alterations of both DNA methylation (5-methylcytosine, 5mC) patterns and chromatin structure are anticipated to be of key importance for the initiation and progression of human cancer [1–3]. Genomic distribution of 5mC undergoes dramatic transformation during tumorigenesis resulting in aberrant patterns of gene expression due to hypermethylation of promoters of tumour suppressor genes and to hypomethylation of oncogene’s promoters [3, 4]. Thus, malignant transformation is determined by both de novo methylation and demethylation of specific genomic regions [4, 5].

The molecular mechanisms of active DNA demethylation were largely obscure until a number of studies demonstrated that Tet (ten-eleven translocation) proteins (Tet1/2/3) can oxidise 5mC to 5-hydroxymethylcytosine (5hmC), 5-formylcytosine (5fC) and 5-carboxylcytosine (5caC) [6–8]. Remarkably, apart from their potential roles in the regulation of transcription, these oxidised forms of 5mC (oxi-mCs) may also serve as intermediates in active and passive demethylation mechanisms [9–12]. Thus, both 5fC and 5caC can be recognised and excised from DNA by thymine DNA glycosylase (TDG) followed by incorporation of non-modified cytosine into the generated abasic site by the components of base-excision repair (BER) pathway [7, 8, 11]. Despite the putative involvement of the oxi-mCs in the mechanisms of DNA demethylation, the roles of these epigenetic modifications in cancer initiation and progression are currently mostly unclear [13]. However, there is a growing body of experimental evidence suggesting that both oxi-mCs and Tet proteins are important for the processes of malignant transformation [5, 13]. Thus, it is currently widely acknowledged that depletion of 5hmC represents an epigenetic hallmark of a number of human cancers [14–17]. In addition, in our recent study, we, rather unexpectedly, found that 5caC is enriched in a proportion of breast cancers and gliomas [18].

Potential biological functions of Tet-dependent 5mC oxidation have been extensively studied in adult brain tumours during several recent years [19–21]. However, neither the oxi-mCs content nor the expression levels of Tet proteins have been assessed in paediatric brain tumours. Nevertheless, a range of tumour suppressors and other genes involved in cancer pathogenesis are aberrantly methylated in both paediatric medulloblastomas and ependymomas implying that DNA (de)methylation plays important role in initiation and/or progression of these types of cancer [22].

In the present study, we aimed to determine the global levels and nuclear distribution of oxi-mCs as well as the expression of *TET1/2/3* and *TDG* transcripts in tumour cell lines derived from paediatric medulloblastomas and ependymomas.

### Methods

#### Cell lines and cell culture

BXD-1425EPN [23], DKFZ-EP1NS [24] and HeLa cells were cultured in Dulbecco’s modified Eagles medium (DMEM) (Gibco, Life Technologies) supplemented with 10% foetal bovine serum and 1% penicillin/streptomycin. Daoy [25] cells were cultured in MEM/EBSS supplemented with 10% heat-inactivated foetal bovine serum, sodium pyruvate, non-essential amino acids, 2 mL glutamine, 100 g/mL streptomycin and 100 U/mL penicillin. The UW228-3 [26] cell line was cultured in DMEM/F12 supplemented with 10% heat-inactivated foetal bovine serum, 2 mL glutamine, 100 g/mL streptomycin and 100 U/mL penicillin.

#### Immunocytochemistry, immunohistochemistry, confocal microscopy, quantification of the signal intensities and statistical analysis

Immunochemistry, confocal microscopy and generation of 2.5XD intensity plots and intensity profiles were performed as previously described [27]. Anti-5hmC mouse monoclonal (Active Motif, 1:5000 dilution) and anti-5caC rabbit polyclonal (Active Motif, 1:500 dilution) primary antibodies were used for immunochemistry. Peroxidase-conjugated anti-rabbit secondary antibody (Dako) and the tyramide signal enhancement system (PerkinElmer, 1:200 dilution, 2 min of incubation with tyramide) were employed for 5caC detection. 5hmC was visualised using 555-conjugated secondary antibody (Alexafluor). Control staining without primary antibody produced no detectable signal. Paraffin-embedded formaldehyde-fixed 12.5 dpc murine embryonic tissue was used for 5caC/5hmC immunostaining of embryonic brain cells. For quantification of the 5hmC and 5caC signal intensities in multiple cells, mean values of the average intensities of eight intensity profiles were calculated for each cell line or, for DKFZ-EP1NS cells, for the populations of 5caC-positive and 5caC-negative cells. Statistical significance was determined by two-tailed *t* test after assessing the variance with *F* test.

#### Gene expression analysis

Expression of *TET1/2/3* and *TDG* transcripts was analysed by quantitative PCR according to standard procedures. Gene expression was normalised by comparison to levels of *GAPDH* gene expression. The following primers were used:

- *TET1*: CTTGGTATGAGTGGGAGTG and GAGCATTAAAGGTAGCAATTG;
- *TET2*: GCAAGATCTTCTTCACAG and GCATGGTTATGTATCAAGTA;
- *TET3*: CTCTGAAGTCAGAGGAGAA and GTCCAGGAAGTTGTGTTC;
- *TDG*: CAGCTATTCCCTTCAGCA and GGAACTTCTTCTGGCATTTG;
- *GAPDH*: GATGCTGGCGCTGAGTACG and GCAGAGATGATGACCCTTTTGG.

### Results

**Fig. 1:**
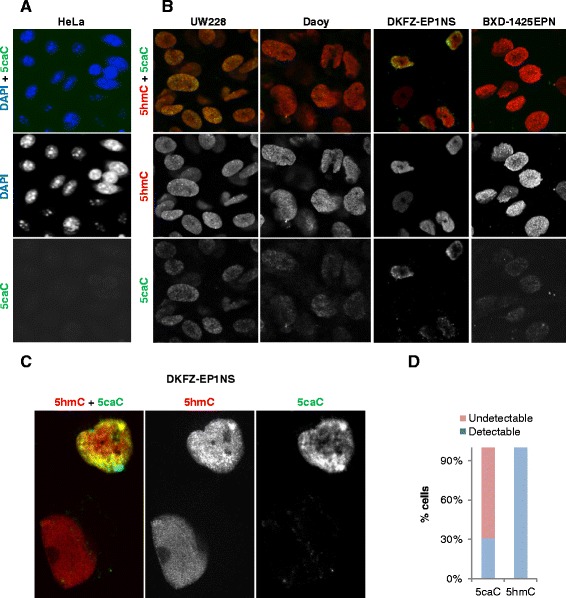
Paediatric medulloblastoma and ependymoma cell lines exhibit immunochemically detectable levels of 5hmC and 5caC. **a**, **b** Co-detection of 5caC with DAPI (**a**) and 5hmC (**b**) in HeLa cells and indicated paediatric brain tumour cell lines. Merged views and individual channels are shown. The cell cultures were stained in parallel at the same experimental conditions and were imaged at identical settings. **c** Co-immunostaining of 5hmC and 5caC in two representative DKFZ-EP1NS cells with different levels of 5caC signal (designated as detectable for the top nucleus and undetectable for the bottom nucleus) used for the categorization of 5caC staining presented in **d**. **d** Proportions of DKFZ-EP1NS cells with detectable or undetectable levels of 5caC and 5hmC signal

To examine the global levels of oxi-mCs in paediatric brain tumours, we initially performed co-detection of 5hmC with 5caC in two sonic hedgehog (SHH) pathway-activated medulloblastoma (UW228-3 and Daoy) and two ependymoma (BXD-1425EPN and DKFZ-EP1NS) cell lines using a protocol for sensitive immunostaining of modified forms of cytosine that we previously developed and validated by mass spectrometry [27]. Unlike in HeLa cells where 5caC was undetectable by immunochemistry under our experimental conditions (Fig. 1a), we observed non-negligible levels of both 5hmC and 5caC immunostaining in all the tested medulloblastoma and ependymoma cell lines (Fig. 1b). Remarkably, the intensity of the 5caC staining differed rather extensively between the paediatric brain tumour cell lines (Fig. 1b). Moreover, whereas most of the BXD-1425EPN, UW228-3 and Daoy cells exhibited similar levels of 5caC signal, the intensity of 5caC staining varied from strong (30% of cells in culture) to undetectable (70% of cells) in DKFZ-EP1NS cells (Fig. 1c, d).

**Fig. 2:**
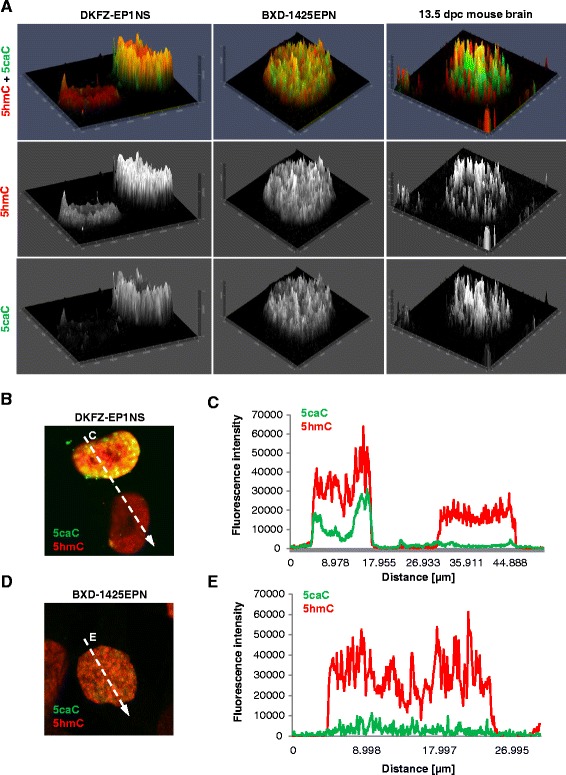
Nuclear localization of 5caC and 5hmC in ependymoma cell lines. **a** 2.5XD 5caC and 5hmC signal intensity plots of the nuclei of two DKFZ-EP1NS cells with different levels of 5caC staining and a representative BXD-1425EPN nucleus compared with 2.5XD 5caC/5hmC signal intensity plot of a representative nucleus of a 13.5 dpc mouse brain cell. Merged views and individual channels are shown. **b**–**e** Merged views of the confocal images of 5caC and 5hmC immunostaining in representative nuclei of DKFZ-EP1NS and BXD-1425EPN cells (**b**, **d**) with *arrows* designating nuclear regions used for generation of the signal intensity profiles shown in **c** and **e**

**Fig. 3:**
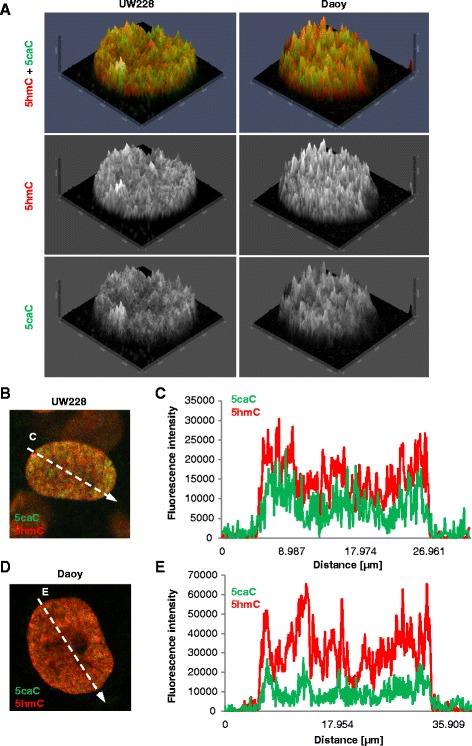
Nuclear localization of 5caC and 5hmC in medulloblastoma cell lines. **a** 2.5XD 5caC and 5hmC signal intensity plots of the representative nuclei of UW228-3 and Daoy cells. Merged views and individual channels are shown. **b**–**e** Merged views of the confocal images of 5caC and 5hmC immunostaining in representative nuclei of UW228-3 and Daoy cells (**b**, **d**) with *arrows* designating nuclear regions used for generation of the signal intensity profiles shown in **c** and **e**

We previously characterised the dynamics of 5caC levels in mouse embryonic brain tissue and showed that this mark transiently accumulates during lineage specification of neural stem cells peaking around 12.5–13.5 days post coitum (dpc) [27]. Interestingly, we found that 5hmC and 5caC were distributed in a semi-overlapping manner in the majority of 13.5 dpc murine brain cells, which suggested that the oxidation of 5mC to 5caC is limited to specific genomic regions in these cells [27]. Based on these results, we decided to compare the nuclear distribution of 5hmC and 5caC in paediatric brain tumours with that of the cells of the murine embryonic brain at 13.5 dpc stage. Analysis of our confocal images revealed that, unlike in the cells of mouse embryonic brain, 5hmC and 5caC display high degrees of spatial overlap in the nuclei of the medulloblastoma and ependymoma cell lines we tested (Figs. 2a–e and 3a–e). Thus, 2.5XD signal intensity profiles were virtually identical for 5hmC and 5caC in the paediatric brain tumour cell lines (Figs. 2a and 3a); and signal intensity profiles for both modifications were mimicking each other in ependymoma and medulloblastoma cells (Figs. 2b–e and 3b–e), suggesting that 5mC oxidation to 5caC occurs genome-widely in these cell lines.

**Fig. 4:**
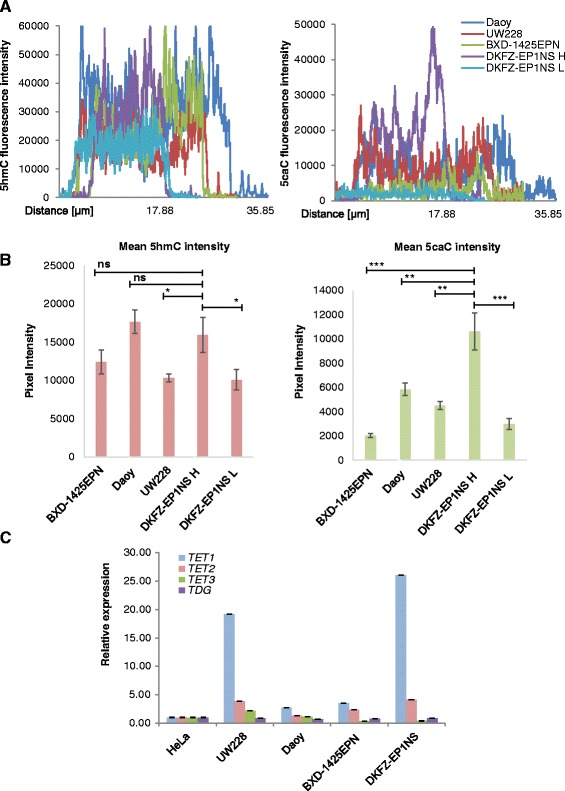
5caC enrichment correlates with elevated levels of TET1 transcript in paediatric brain tumour cell lines. **a** Overlays of 5hmC (*left*) and 5caC (*right*) signal intensity profiles for representative nuclear regions of the indicated paediatric brain tumour cells. Signal intensity profiles for DKFZ-EP1NS cells with high/detectable (DKFZ-EP1NS H) and low/undetectable (DKFZ-EP1NS L) 5caC signals are shown separately. **b** Quantification of 5hmC and 5caC signals in the indicated ependymoma and medulloblastoma cell lines. Mean values of the average intensities of eight signal intensity profiles for each cell line/population are shown. *DKFZ-EP1NS H* cells with high/detectable 5caC, *DKFZ-EP1NS L* cells with low/undetectable 5caC. ***p < 0.001; **p < 0.01; *p < 0.05; *ns* not significant. **c** Relative expression of Tet1*/2/3* and *TDG* transcripts in the indicated paediatric brain tumour cell lines and HeLa cells. Experimental error is shown as SEM

Next, we attempted to compare the intensities of 5hmC and 5caC signals between the four tested paediatric brain tumour cell lines employing analysis of the individual signal intensity profiles and quantification of the staining intensities in multiple cells (Fig. 4a, b). Both approaches demonstrated that, whereas the levels of 5hmC signal were comparable between all the cell lines, 5caC signal in a subpopulation of DKFZ-EP1NS cells positive for this modification (DKFZ-EP1NS H) was significantly higher (*p* < 0.01 to *p* < 0.001) compared with other paediatric brain tumour cell lines (Fig. 4b). Importantly, the levels of 5caC immunostaining did not correlate with 5hmC signal intensity in the SHH medulloblastoma and ependymoma cell lines (Fig. 4b). To get an insight into potential molecular mechanisms for the 5caC enrichment in medulloblastoma and ependymoma cells, we examined the levels of *TET1*/2/3 and *TDG* transcripts in the four paediatric brain tumour cell lines and HeLa cells. These experiments revealed that neither *TDG* nor *TET*3 expression was substantially altering between all the five tested cell lines (Fig. 4c). In contrast, expression of *TET2* and *TET1* was generally higher (e.g. 2.37-fold for BXD-1425EPN and 4.14-fold for DKFZ-EP1NS cells for *TET2*) in the brain tumour cell lines compared with HeLa cells. However, the levels of *TET1* transcript exhibited the most dramatic increase in DKFZ-EP1NS and UW228-3 cells differing from HeLa in 26- and 19-fold correspondingly (Fig. 4c). Remarkably, the elevated levels of *TET1* expression in DKFZ-EP1NS and UW228-3 corresponded to strong 5caC enrichment we observed in these cells.

### Discussion

In a recently published review of TET1 functions in cancer, the authors came to conclusion that this protein has a dual role in tumorigenesis [5]. Thus, according to a number of studies, *TET1* expression is decreased in different types of malignant tissue [16, 28, 29]. Moreover, suppression of TET1 expression was reported to be associated with facilitated cell invasion and metastasis [30, 31] and even to play a critical role in KRAS-induced tumour transformation [32]. Contrasting with these reports, there is experimental evidence that TET1 acts as an oncogene in MLL-rearranged leukaemia and breast tumour malignancies [33, 34]. In this context, our observation that the levels of *TET1* transcript are elevated in medulloblastoma and ependymoma cells may imply that this protein is involved in pathogenesis of the paediatric brain tumours via demethylation of the regulatory elements of the oncogenes promoting initiation and/or progression of these types of cancer.

Medulloblastoma and ependymoma represent the two most common forms of malignant paediatric brain tumours. Both tumour types have recently been categorised into clinically relevant molecular subgroups [35, 36], which can be recapitulated by methylation analyses supporting the hypothesis that epigenetic drivers may play a key role in pathogenesis of these tumour types [37, 38]. Thus, the current classification of medulloblastomas include *Wnt*, sonic hedgehog (SHH) and group 3 and 4 subtypes [39, 40]. The pairs of cell lines used in this study represent the most aggressive subgroups of each tumour type that respond poorly to current therapeutic approaches. UW228-3 and Daoy are both SHH pathway-activated lines harbouring a mutant *TP53* gene (SHH-activated, *TP53* mutant) [39]. BXD-1425EPN and DKFZ-EP1NS on the other hand represent a subgroup of ependymomas that carry a *C11orf95-RELA* fusion oncogene which results in activation of the NF-κB signalling pathway [40]. Remarkably, TP53 function has also recently been shown to be abrogated in the majority of *RELA* ependymomas where it is associated with particularly poor outcome [41]. In addition, both ependymomas and SHH medulloblastomas have been demonstrated to maintain a population of stem-like cells [42, 43]. These cells express cancer stem cell and neural stem cell markers CD133 and Nestin [42, 44–46]. Highly tumorigenic and metastatic ependymoma cell line DKFZ-EP1NS, which demonstrates in vivo primary tumour recapitulation ability in orthotopic xenograft models, expresses both these stem cell markers together with CD15 and ALDH [24]. Importantly, expression of CD15 and ALDH is also a feature shared by aggressive SHH medulloblastoma cell lines UW228-3 and Daoy [24, 47]. Correspondingly, deregulation of signalling pathways important for embryonic brain development (e.g. SHH, Wnt and Notch pathways) appears to be a hallmark of both ependymomas and medulloblastomas and to play essential role in pathogenesis of these tumours [22]. Likewise, in line with anticipated significance of Tet proteins for neuro- and gliogenesis [27], the aberrantly increased *TET1*-dependent 5mC oxidation may represent one of the epigenetic signatures of these cancers reflecting their likely neural progenitor/stem cell origin.

Interestingly, although 5caC enrichment corresponded to remarkably high levels of *TET1* mRNA in DKFZ-EP1NS and UW228-3 cell lines in our experiments, in Daoy cells, high intensities of both 5hmC and 5caC staining were paralleled by levels of *TET1/2/3* and *TDG* transcripts comparable with those in HeLa cells where 5caC was not detectable by immunochemistry under our conditions. This suggests that either specific post-transcriptional mechanisms of regulation of *TET1*/2/3 expression may be operative in this cell line or oxi-mCs may be stabilised there due to certain features of DNA methyltransferases, oxi-mCs-interacting proteins and/or components of BER machinery specific for Daoy cells.

Although the presence of 5fC and 5caC in genomic DNA is often perceived as an indication of active TDG-dependent DNA demethylation, a growing body of experimental evidence suggests that all the oxi-mCs may play their own specific roles in gene regulation [27, 48–50]. Thus, developmental dynamics of 5fC is different from that of 5hmC [50], and different oxi-mCs are associated with distinct sets of regulatory sequences in the genome [27, 51]. Moreover, specific groups of candidate “reader” proteins have been identified for each of the oxi-mCs using mass spectrometry-based approaches [52]. Interestingly, the lists of potential “reader” proteins for 5fC and 5caC include a number of transcription factors, chromatin remodelling proteins and histone-modifying enzymes [52]. Therefore, our data revealing the genome-wide 5caC enrichment in UW228-3, Daoy and a subpopulation of DKFZ-EP1NS cells suggest that the presence of this modification in regulatory genomic regions may not only be linked with their demethylation but also affect transcriptional activity of the corresponding genes in these cells via 5caC-dependent recruitment of transcriptional factors or chromatin modifying complexes, contributing to the malignant phenotypes of the paediatric brain tumour cell lines.

Further studies should evaluate functional significance of increased levels of TET1 and 5caC in medulloblastoma and ependymoma providing new information on the pathogenesis and potentially leading to development of novel targets for therapy of these brain tumours. In addition, UW228-3, Daoy and DKFZ-EP1NS cells may represent a suitable experimental model to study the molecular mechanisms of Tet-dependent 5mC oxidation and potential roles of oxi-mCs in transcriptional regulation.

## Abbreviations

Tet1: Ten-eleven translocation protein 1
